# *Pseudomonas aeruginosa* Dps (PA0962) Functions in H_2_O_2_ Mediated Oxidative Stress Defense and Exhibits In Vitro DNA Cleaving Activity

**DOI:** 10.3390/ijms24054669

**Published:** 2023-02-28

**Authors:** Nimesha Rajapaksha, Anabel Soldano, Huili Yao, Fabrizio Donnarumma, Maithri M. Kashipathy, Steve Seibold, Kevin P. Battaile, Scott Lovell, Mario Rivera

**Affiliations:** 1 Department of Chemistry, Louisiana State University, 232 Choppin Hall, Baton Rouge, LA 70803, USA; 2 Protein Structure and X-ray Crystallography Laboratory, University of Kansas, 2034 Becker Dr., Lawrence, KS 66047, USA; 3 NYX, New York Structural Biology Center, Upton, NY 11973, USA

**Keywords:** Dps, mini ferritin, ferritin, iron metabolism, oxidative stress, peroxide toxicity, exonuclease, DNA cleaving activity

## Abstract

We report the structural, biochemical, and functional characterization of the product of gene PA0962 from *Pseudomonas aeruginosa* PAO1. The protein, termed Pa Dps, adopts the Dps subunit fold and oligomerizes into a nearly spherical 12-mer quaternary structure at pH 6.0 or in the presence of divalent cations at neutral pH and above. The 12-Mer Pa Dps contains two di-iron centers at the interface of each subunit dimer, coordinated by conserved His, Glu, and Asp residues. In vitro, the di-iron centers catalyze the oxidation of Fe^2+^ utilizing H_2_O_2_ (not O_2_) as an oxidant, suggesting Pa Dps functions to aid *P. aeruginosa* to survive H_2_O_2_-mediated oxidative stress. In agreement, a *P. aeruginosa* Δ*dps* mutant is significantly more susceptible to H_2_O_2_ than the parent strain. The Pa Dps structure harbors a novel network of Tyr residues at the interface of each subunit dimer between the two di-iron centers, which captures radicals generated during Fe^2+^ oxidation at the ferroxidase centers and forms di-tyrosine linkages, thus effectively trapping the radicals within the Dps shell. Surprisingly, incubating Pa Dps and DNA revealed unprecedented DNA cleaving activity that is independent of H_2_O_2_ or O_2_ but requires divalent cations and 12-mer Pa Dps.

## 1. Introduction

The requirement of iron as a nutrient, the reactivity of Fe^2+^ toward O_2_ and H_2_O_2_, and the insolubility of Fe^3+^ at biologically compatible pH present significant challenges to bacterial cells, which are managed by the iron homeostasis machinery (iron uptake, storage, and utilization) [1,2]. Iron storage proteins enable iron homeostasis by allowing the accumulation of intracellular iron while simultaneously ameliorating the toxicity mediated by the extreme insolubility of Fe^3+^ and the oxidative stress resulting from uncontrolled Fe^2+^/Fe^3+^ redox cycling. In many bacteria, iron is stored in three types of protein, ferritin, bacterioferritin, and possibly in DNA binding protein from starved cells (Dps). *Pseudomonas aeruginosa*, a metabolically versatile Gram-negative bacterium that is often present in environments affected by human activities [3], can cause acute and chronic infections in patients with catheters, diabetic foot ulcers, burn wounds, and surgical incisions [4,5]. The interactions between *P. aeruginosa* and the host generate environments with high concentrations of reactive oxygen species, which the bacterium can survive because of multiple antioxidant systems, including iron storage proteins. The *P. aeruginosa* genome [6] harbors at least four genes encoding proteins thought to be involved in iron storage: *ftnA* (PA4235) codes bacterial ferritin (FtnA) [7], *bfrB* (PA3531) codes a bacterioferritin (BfrB) [8,9], PA0962 codes a probable Dps, and PA4880 codes a probable bacterioferritin.

Our laboratory has been focused on understanding the function of iron storage proteins in *Pseudomonas aeruginosa* [10,11]. We have shown that this organism utilizes a heteropolymeric bacterioferritin (Bfr) molecule assembled from FtnA and BfrB subunits to store iron, with subunit composition dependent on the environmental availability of O_2_ [12]. Mobilization of Fe^3+^ stored in Bfr requires electron transfer from a ferredoxin (Bfd) within a bimolecular complex [13]. A blockade of the Bfr-Bfd complex in *P. aeruginosa* cells creates a lesion in the iron homeostatic machinery, which leads to irreversible accumulation of iron in Bfr and intracellular iron limitation [14]. Beyond generating iron starvation, this lesion in the iron metabolism of *P. aeruginosa* adversely affects carbon and sulfur metabolism [15] and the ability to maintain the biofilm lifestyle [16]. These fundamental findings motivated the discovery of small molecules for inhibiting the Bfr–Bfd complex, which can kill biofilm-entrenched cells [17,18], thus suggesting iron storage/mobilization as a target for antibiotic development. 

Despite the significant progress in elucidating the structure and function of Bfr in *P. aeruginosa* iron metabolism, nothing is known about the structure and function of the two other proteins thought to participate in iron storage, the product of gene PA0962, a probable Dps, and the product of the PA4880 gene, a putative bacterioferritin. Herein we concentrate on elucidating the structure and function of the protein coded by the PA0962 gene, a putative Dps (DNA binding protein under starvation). Dps, which was discovered from starving *E. coli* cells, is thought to bind DNA to protect it from stressors such as peroxide-induced oxidative damage [19,20], nutrient limitation, thermal stress, and UV radiation [21]. The structure of *E. coli* Dps (Ec Dps) showed a 12-mer assembly of identical subunits, where the subunit fold has structural homology to ferritin [10,22,23]. In comparison to ferritin and bacterioferritin, which are spherical and hollow assemblies of 24 subunits with internal and external diameters of ~8 nm and ~12 nm, respectively [10], Dps form 12-mer assemblies with internal and external diameters of ~5 nm and ~9 nm, respectively [23,24]. Two ferroxidase centers are located at the interface of subunit dimers, such that a 12-mer Dps harbors 12 ferroxidase sites [25]. 

Herein we report that the structure of the protein coded by gene PA0962 reveals a genuine Dps fold; thus, the protein is termed *Pseudomonas aeruginosa* Dps (Pa Dps). Assembly of Pa Dps into a 12-mer structure in solution is conditioned by the presence of divalent ions at neutral and higher pH but occurs spontaneously at acidic pH. Pa Dps exhibits H_2_O_2_-dependent ferroxidase activity in vitro, and it contributes to the survival of *P. aeruginosa* cells challenged with H_2_O_2_-mediated oxidative stress. Surprisingly, Pa Dps exhibits endonuclease activity in vitro.

## 2. Results and Discussion

### 2.1. Pa Dps Oligomerization in Solution

**Figure 1:**
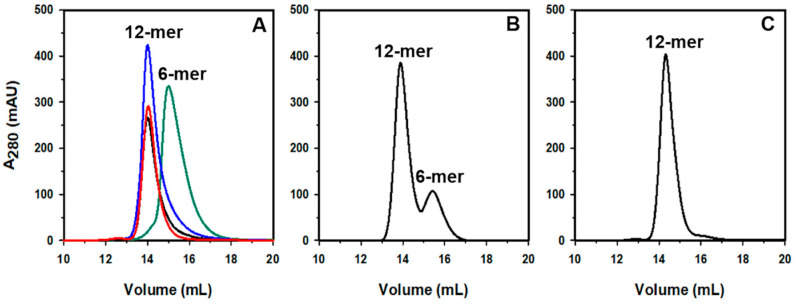
The multimeric assembly of Pa Dps is affected by divalent metal ions or pH. Pa Dps samples were equilibrated in distinct buffer conditions and then chromatographed on a calibrated Superdex S200 column equilibrated and eluted with the same buffer: (**A**) 75 mM Tris pH 7.5 (green); 75 mM Tris pH 7.5 and 1 mM Mg^2+^ (blue), 75 mM Tris pH 7.5 and 0.5 mM Ca^2+^ (black), 75 mM Tris pH 7.5 and 0.5 mM Mn^2+^ (red); (**B**) 75 mM Bis-Tris pH 6.5. (**C**) 75 mM Bis-Tris pH 6.0.

The oligomerization state of purified Pa Dps was assessed on a calibrated Superdex S200 size exclusion column equilibrated and eluted with 75 mM Tris, pH 7.5. The elution volume (V_e_) suggests a Dps hexamer (Figure 1A). If Pa Dps is equilibrated in 75 mM Tris, pH 7.5 containing Mg^2+^, Ca^2+^, or Mn^2+^ and subsequently loaded onto a Superdex S200 column equilibrated and eluted with the same buffer, the elution volume shifts to that predicted for a dodecameric Dps (Figure 1A). Removal of divalent cations, followed by loading onto the Superdex S200 column equilibrated with 75 mM Tris pH 7.5, reverts the V_e_ to that corresponding to hexamer. The assembly of 12-mer Pa Dps is also influenced by pH: at pH 6.5, the protein elutes in two peaks corresponding to 12-mer and 6-mer assemblies (Figure 1B), whereas at pH 6.0 Pa Dps elutes as 12-mer (Figure 1C).

### 2.2. Pa Dps X-ray Crystal Structure 

**Figure 2:**
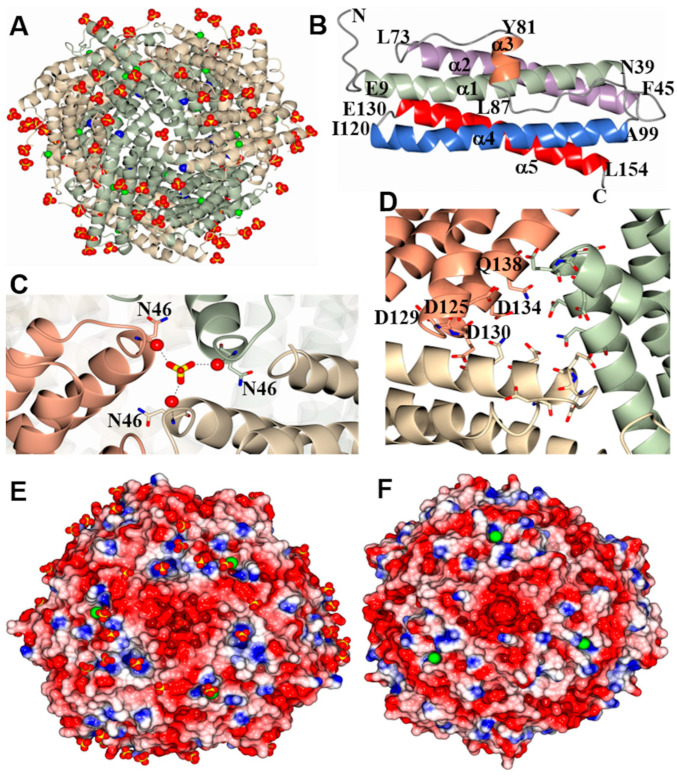
Structure of Pa Dps: (**A**) View of a Pa Dps dodecamer model obtained from Dps-o crystals. Green/wheat ribbons render the dodecamer subunits. Sodium and chloride ions are in blue and green spheres, respectively, and sulfate ions are shown as yellow/red spheres. (**B**) View of a Pa Dps-o subunit illustrating secondary structure elements. (**C**) View of one of the A-type three-fold pores formed by the convergence of three subunits (wheat, salmon, and green); the sulfate ion is in yellow/red sticks, and water molecules are depicted as red spheres. (**D**) View of one of the B-type three-fold pores (wheat, salmon, green) and negatively charged residues lining the pore interior (residues are noted for one of the subunits). Electrostatic surface representation of an A-type pore (**E**) and a B-type (ferritin-like) pore (**F**) in Pa Dps-o; the sulfate ions (yellow/red) and chloride ions (green) are rendered as spheres. Units are in volts from −0.5 (red) to +0.5 blue.

The screening of crystallization conditions produced two crystal forms, prismatic crystals of a primitive orthorhombic form (Dps-o) and primitive cubic lattice crystals (Dps-c). Structure solution and refinement (Table S1) resulted in a Dps-o model containing 12 subunits in the asymmetric unit. One sodium ion and two chloride ions bind to each subunit, and 82 sulfate ions (acquired from the crystallization solution) are associated with the 12-mer (Figure 2A). The 12-mer assembly forms a nearly spherical shell (23-point group symmetry, ~9 nm diameter), which surrounds a hollow interior cavity (~4 nm diameter). Each of the subunits adopts the characteristic Dps fold, which starts with a short stretch of random coil N-terminal residues (M1–G8) that leads onto a 4-helix bundle where helices α1 and α2 are connected to helices α4 and α5 by a long loop which harbors a two-turn helix, α3 (Figure 2B). The Cl^−^ associated with each subunit is surrounded by residues in the long loop and residues in α5 (Figure S1A). The Na^+^ is located at inter-subunit sites coordinated by H37 from one subunit and D64 and E68 from the second subunit (Figure S1B); only five of the twelve D64 residues coordinate a sodium ion in the structure, and these may be interchangeable with water molecules.

A total of four three-fold axes traverse the 12-mer Dps, each passing through two trimeric interfaces (three-fold pores). Each pair of three-fold pores along a three-fold axis exhibits distinct microenvironments, giving rise to two types of three-fold pores, termed here A and B. In the A-type three-fold pores, residues near the turn separating α1 and α2 form the pore trimeric interface, which is defined by P43, N46, T47, M44, and E100, and in the model contains an SO_4_^2−^, which forms water-mediated contacts with the backbone N-atom of N46 (Figure 2C). The B-type three-fold pores (also known as ferritin-like pores), which are at trimeric interface regions near the turns separating α4 and α5, are defined by D125, D129, D130, and D134 (Figure 2D). The electrostatic surface of the A-type three-fold pore interior is positive, which probably stabilizes the SO_4_^2−^ ion, while the electrostatic surface in the immediate vicinity of the pore exterior is negative and is surrounded by three patches of positive potential where the SO_4_^2−^ and Cl^−^ ions in the structure nest (Figure 2E). In comparison, a strong negative electrostatic surface dominates the B-type three-fold pore interior, while the exterior diameter on the pore surface is surrounded by a patch of neutral electrostatic potential (Figure 2F). 

**Figure 3:**
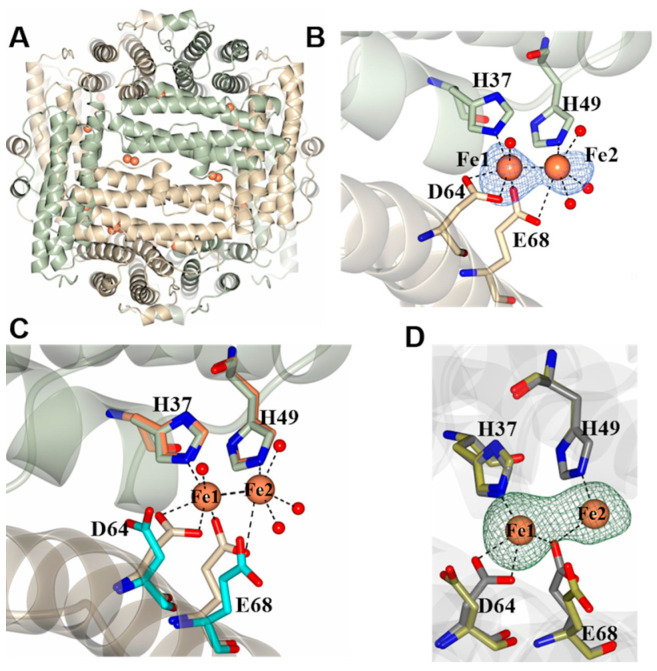
Iron binding sites in Dps: (**A**) View of the two identical di-iron binding sites that are present at the interface of subunit dimers in Dps-c-Fe; the iron ions are rendered as orange spheres, and the subunits in each subunit dimer as green and gray ribbons. (**B**) Expanded view illustrating the coordination of the iron ions at a di-iron site. The phased anomalous difference map (blue mesh) is contoured at 3σ, and the water molecules are rendered as red spheres. (**C**) Superposition of apo- and Fe-bound Pa Dps-c structures; Pa Dps-c (orange/cyan) and Pa Dps-c-Fe (wheat/green). (**D**) Superposition of apo- and Fe-bound Pa Dps-o structures; Pa Dps-o (green) and Pa Dps-o-Fe (gray). The superpositions illustrate the conformational changes incurred upon iron binding. The green mesh in panel (**D**) is the Fo-Fc difference electron density map contoured at 3σ.

The Dps-c structure is nearly identical to the Dps-o structure, as indicated by the 0.38 Å RMSD deviation observed on superposing the Cα atoms in the dodecamers (1832 residues). To identify the ferroxidase center ligands, the Dps-o and Dps-c crystals were soaked for 10 min in FeCl_2_ solution prior to X-ray diffraction data acquisition. The corresponding structures are termed Dps-o-Fe and Dps-c-Fe, respectively. Although the findings described below are in reference to the Dps-c-Fe structure, the observations made with both structures are very similar. These experiments allowed the identification of twelve di-iron ferroxidase sites located between subunit dimers, with each subunit dimer containing two di-iron binding sites related to one another by two-fold symmetry (Figure 3A). The two Fe ions in each di-Fe site (Fe1 and Fe2) are 2.59 Å apart and are coordinated by two conserved His side chains stemming from one subunit (H37 and H49) and two conserved carboxylate side chains from the other (D64 and E68); water molecules complete an octahedral coordination field for each iron (Figure 3B). The presence of iron in the model was corroborated by an anomalous difference in electron density (blue mesh) from data collected at a wavelength of 1.5498 Å. The electron density at Fe1 is more intense than that at Fe2, and the corresponding average refined occupancies are 0.86 and 0.64, respectively. Note that Fe1 is in the same site as the sodium ions observed in the Dps-o structure (Figure S1B). Superposition of the Dps-c and Dps-c-Fe structures (Figure 3C) or Dps-o- and Dps-o-Fe (Figure 3D) shows that only small rearrangements of D64 and E68 side chains are required to bind the iron ions in the ferroxidase centers of Pa Dps.

**Figure 4:**
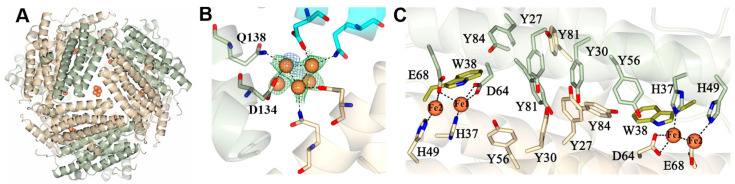
(**A**) View of a cluster of iron ions in a B-type 3-fold pore. (**B**) The side chains of D134 and Q138 in each subunit interact with the iron ions. The phased anomalous difference map (blue mesh) is contoured at 3σ, and 2Fo-Fc map (green mesh) is contoured at 1σ. (**C**) View of a C2 symmetry-related dimeric interface in Pa Dps illustrating the two ferroxidase centers and the network of Tyr residues. The two subunits and associated Tyr residues are rendered in green and wheat, and the conserved Trp in both subunits is rendered in yellow. The dotted lines highlight coordination of iron by ferroxidase center ligands.

The Dps-c-Fe structure also showed patches of anomalous difference density and Fo-Fc and 2Fo-Fc electron density within B-type three-fold pores, which were modeled as clusters of iron ions where three of the ions are coordinated by D134 and Q138 near the interior cavity (Figure 4A,B). In this context, it is of note that the B-type pores are thought to enable the traffic of iron in and out of the Dps cavity [26]. Inspection of the type-A three-fold pores reveals the presence of a HEPES molecule in each of the pores (Figure S2); note that the hydroxyethyl moiety of each HEPES molecule is disordered. The SO_3_^−^ moiety of HEPES, which makes water mediated contacts with the backbone N-H of N46 and with the side chains of T47 (which adopt two alternate conformations), is in a location nearly identical to that occupied by SO_4_^2−^ in the Dps-o structure (Figure 2C). It is, therefore, possible that the A-type three-fold pores in Pa Dps enable traffic of anions, such as sulfate or phosphate, across the protein shell. As has been proposed based on similar observations made with Bfr from *P. aeruginosa*, traffic of anions across the protein shell is important to balance the charge of Fe^3+^ in the interior cavity [9,10,27].

A striking feature in the structure of Pa Dps is a network of ten Tyr side chains located at the interface of subunit dimers, halfway between the two ferroxidase centers (Figure 4C). A conserved Trp (W38) is also present, located approximately 3Å from Fe1. The large number of Tyr residues composing the network in Pa Dps is, to the best of our knowledge, unique. In Dps from *Listeria innocua* (Li Dps), the conserved Trp (W32) and a Tyr residue (Y50) form a less extensive network which has been implicated in the trapping of hydroxyl radicals and preventing Fenton-mediated oxidative damage [28]. As shown below, the network of Tyr side chains in Pa Dps functions similarly.

### 2.3. Pa Dps in Complex with Mn^2+^

**Figure 5:**
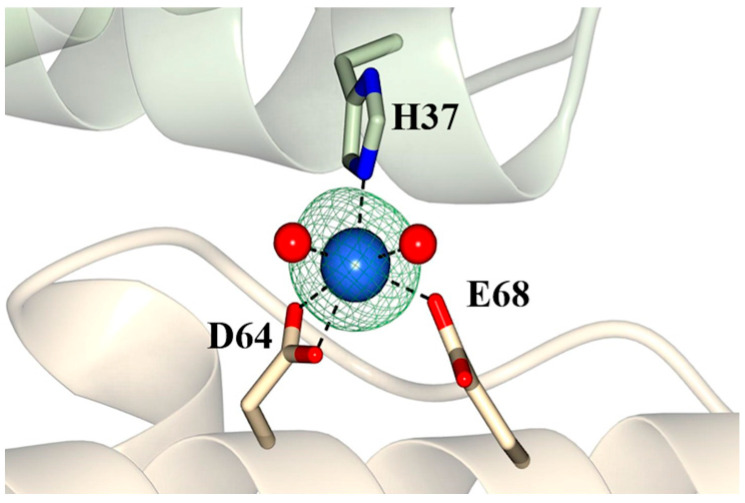
Mn (blue sphere) coordinated by ferroxidase ligands H37 (subunit 1) and D64 and E68 (subunit 2) occupies the same site as Na^+^ in the Dps-o structure (Figure S1) and Fe1 in the Dps-c-Fe and Dps-o-Fe structures (Figure 3). The Fo-Fc omit map (green mesh) is contoured at 5σ.

Pa Dps assembles into a 12-mer structure when the buffer (75 mM Tris, pH 7.5) includes Mg^2+^ or Mn^2+^. The Dps-o structure revealed the presence of a Na^+^ coordinated by ferroxidase center ligands H37, D64, and E68 (Figure S1B). The Na^+^ occupies the same site as Fe1 in the di-Fe center of the Dps-c-Fe and Dps-o-Fe structures, which is coordinated by the same three ligands (Figure 3). In comparison, Fe2 is coordinated by only two protein-provided ligands, H49 and E68. Consequently, we hypothesized that the “Fe1-site” might have a higher propensity to bind metal ions and therefore contribute to stabilizing the subunit dimer and possibly the dodecameric structure. To test this idea, we crystallized Pa Dps from a solution containing 0.5 mM MnCl_2_ (Dps-c-Mn). The Dps-c-Mn structure is nearly identical to the Dps-c-Fe structure (RMSD = 0.40 Å, 1834 residues). Inspection of the ferroxidase center ligands shows an Mn atom coordinated by H37, D64, and E68 (Figure 5), occupying the same site as Fe1 in the Dps-c-Fe and Dps-o-Fe structures and Na in the Dps-o structure. These findings support the idea that the “Fe-1” site exhibits a higher propensity to coordinate metal ions than Fe2. In this context, it is also interesting to note that the structures of *Listeria innocua* [29], *Helicobacter pylori* [30], and *Agrobacterium tumefaciens* [31] Dps show iron only in the site equivalent to Fe1 in Pa Dps (Figure S3). 

### 2.4. Pa Catalyzes the Oxidation of Fe^2+^ When the Oxidant Is H_2_O_2_

The presence of iron ions coordinated by conserved ferroxidase center ligands in the X-ray crystal structures of Pa Dps suggested that the protein may catalyze the oxidation of Fe^2+^ in solution and compartmentalize Fe^3+^ in its interior cavity. To test this idea, solutions of 12-mer Pa Dps in 75 mM Bis-Tris (pH 6.5) containing 1 mM Mg^2+^ were titrated with aliquots delivering 50 Fe^2+^ per 12-mer Pa Dps. The experiments were carried out in air with O_2_ as an oxidant or in an anaerobic glove box using H_2_O_2_ as an electron acceptor.

**Figure 6:**
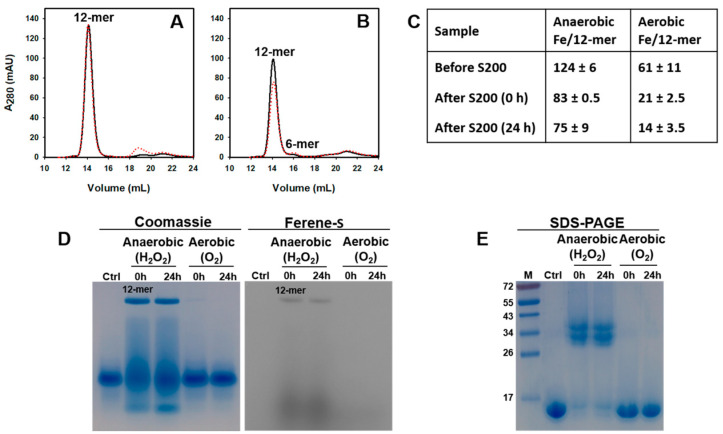
The oxidation of Fe^2+^ at Pa Dps ferroxidase centers requires H_2_O_2_. Pa Dps titrated anaerobically with Fe^2+^ and H_2_O_2_ (**A**) and with Fe^2+^ in air (**B**), was separated in a calibrated Superdex S200 column immediately after the titration (0 h; black trace) and after 24 h at 4 °C (24 h, red). (**C**) Iron associated with 12-mer Pa Dps before and after passage through a Superdex S200 column. Standard deviation is from two independent experiments. (**D**) Twelve-mer Pa Dps eluting from the Superdex S200 column was separated in a native PAGE gel and stained first with Ferene S to visualize iron and then with Coomassie to visualize the protein. (**E**) Twelve-mer Pa Dps eluting from the Superdex S200 column was loaded onto an SDS PAGE gel and stained with Coomassie. Ctrl = 12-mer Pa Dps (75 mM tris, pH 7.5, 1 mM Mg^2+^) not titrated with iron, M = molecular weight marker.

Electronic absorption spectra obtained during a titration carried out in a glove box (Figure S4A) show that the addition of each Fe^2+^ aliquot does not cause spectral changes, consistent with the absence of an oxidant in the solution. The addition of an equivalent of H_2_O_2_ causes the absorbance ca. 320 nm to increase, indicating the formation of Fe^3+^-O moieties. After the addition of the last Fe^2+^ aliquot (total = 300 Fe^2+^/12-mer), the solution was passed through a desalting column, concentrated, and then split in two. A small volume from each sample was used to determine the iron and protein concentrations, and the remainder was loaded onto a Superdex S200 column immediately (0 h) and after 24 h incubation at 4 °C (24 h), followed by an analysis of iron and protein concentrations in the eluting fractions. The chromatograms (Figure 6A) show that Pa Dps remains as 12-mer. The iron and protein concentrations prior to the Superdex S200 column indicate that Pa Dps contained ~41% of the 300 Fe ions/12-mer delivered (~123 Fe). Passage through the Superdex S200 column resulted in the loss of ~40 Fe ions/12-mer (Figure 6C), which may have been dynamically associated with ferroxidase centers, while the remaining ~83 Fe ions/12-mer are likely compartmentalized in the Pa Dps cavity.

When similar experiments were conducted by titrating Pa Dps in air, the oxidation of Fe^2+^ occurred after the delivery of each aliquot (Figure S4B). The fractions eluting from the Superdex S200 column (Figure 6B) indicate that Pa Dps is present as a 12-mer immediately after the titration, but a small proportion dissociates into hexamers after 24 h. The Fe and protein concentrations in the eluting fractions containing 12-mer indicate the presence of ~60 Fe ions/12-mer prior to passage through the Superdex S200 column, but only 20 Fe ion/12-mer remain associated Pa Dps after the column (Figure 6C). These observations suggest that most of the iron associated with Pa Dps is not compartmentalized in the interior cavity; instead, it may be coordinated by ferroxidase center ligands and perhaps other transient sites on the 12-mer protein. Hence, the observations suggest that when O_2_ is the oxidant, the majority of Fe^2+^ is adventitiously oxidized by dissolved O_2_, giving rise to Fe^3+^ that binds Pa Dps but is not internalized into the core.

To further explore the idea that the oxidation of Fe^2+^ with H_2_O_2_ occurs at ferroxidase centers and elicits Fe^3+^ compartmentalization in Pa Dps, whereas oxidation of Fe^2+^ with O_2_ is adventitious, the fractions eluting from the Superdex S200 column were loaded onto a native PAGE gel. Staining first with Ferene S and then with Coomassie allows visualization of iron and protein (Figure 6D). The samples from the anaerobic titration of Pa Dps with Fe and H_2_O_2_ exhibit a band corresponding to 12-mer Pa Dps, which corroborates iron compartmentalization. The streak, most evident in the Coomassie-stained gel, suggests lower oligomerization states, which are not observed in fractions eluting from the S200 column (Figure 6A). The lower oligomerization states in the gel are a consequence of 12-mer disassembly due to the absence of divalent metal ions in the native gel and electrophoresis buffer. The fact that 12-mer Pa Dps disassembles in the PAGE gel is also evident in the control lane (ctrl), which, despite having been loaded with 12-mer Pa Dps, does not show a 12-mer band. In comparison, the samples from the titration of 12-mer Pa Dps in the presence of O_2_ (Figure 6D) show the conspicuous absence of a 12-mer band, thus supporting the idea that in air Fe^2+^ is oxidized adventitiously by dissolved O_2_.

A similar analysis of the fractions eluting from the Superdex S200 column, this time resorting to SDS PAGE gels, revealed additional insights. The electrophoretic mobility of the Pa Dps sample that had been titrated with iron in the presence of O_2_ (Figure 6E) is identical to that of Pa Dps not titrated with iron (ctrl). In stark contrast, lanes loaded with the sample titrated anaerobically with iron and H_2_O_2_ show prominent bands that migrate similarly to a 34 kDa standard. These bands suggest that hydroxyl radicals are produced on reacting Pa Dps with Fe^2+^ and H_2_O_2_, which elicit the formation of crosslinked Pa Dps dimers. As shown below, the covalent crosslinks involve the network of Tyr residues depicted in Figure 4C. 

### 2.5. A Network of Tyr Residues Traps Hydroxyl Radical within the Protein Shell

**Figure 7:**
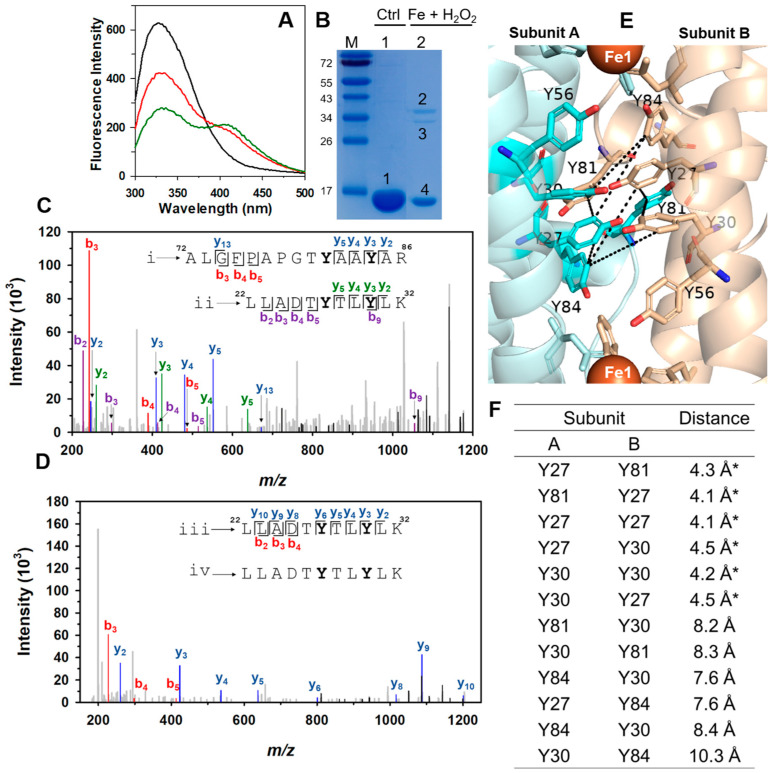
Anaerobic oxidation of Fe^2+^ with H_2_O_2_ leads to Y-Y crosslinked Pa Dps: (**A**) Fluorescence spectra (280 nm excitation) of 12-mer Pa Dps (1 μM) in 75 mM Bis-Tris (pH 6.5) containing 1 mM Mg^2+^ before (black) and after the addition of two aliquots, each delivering 24 Fe^2+^/12-mer and 1 equivalent of H_2_O_2_ (red and green, respectively). (**B**) SDS PAGE gel loaded with Pa Dps (ctrl) and Pa Dps titrated with a total of 48 Fe^2+^/12-mer and one equivalent of H_2_O_2_. M = molecular weight marker. (**C**,**D**) Annotated ESI-MS/MS spectra of Y-Y crosslinked peptides i–ii and iii–iv, respectively. Band 2 in the gel contained crosslinked peptide i–ii, and band 3 contained peptides i–ii and iii–iv. (**E**) Residues implicated in possible Y-Y crosslinks. (**F**) Distances measured from an o-phenol carbon in one Tyr to the closest o-phenol carbon in the accompanying Tyr. The * denotes probable crosslinks.

To investigate the nature of the crosslinked dimers that form when Pa Dps is titrated with Fe^2+^ and H_2_O_2_ under anaerobic conditions, we resorted to fluorescence spectroscopy and mass spectrometry. To this end, Pa Dps was titrated anaerobically with two aliquots, each delivering 24 Fe^2+^/12-mer and one equivalent of H_2_O_2_. The fluorescence spectra (Figure 7A and Figure S5) obtained after the addition of each aliquot show a decrease in the intensity of the band ca. 330 nm and the appearance and growth of a band ca. 410 nm. These observations are consistent with the formation of crosslinked di-tyrosine (Y-Y) moieties [32,33]. Loading the resultant solution onto an SDS PAGE gel revealed the expected ~16 kDa band corresponding to a Pa Dps subunit (band 4) and two low-intensity bands (2 and 3) at a position consistent with a crosslinked dimer of Pa Dps subunits (Figure 7B). Bands 1, 2, and 3 were excised from the gel, and the proteins were subjected to proteolytic digestion. Analysis by high-resolution LC-MS/MS revealed the presence of Y-Y crosslinked peptides in bands 2 and 3 (see below). The reasons for the different electrophoretic mobility of bands 2 and 3 are not known with certainty, but it is possible that distinct Y-Y links and/or oxidative modifications (e.g., Met oxidation) are at play.

Figure 7C,D depict the annotated tandem mass spectra and graphical representation of two Y-Y crosslinked peptides obtained from bands 2 and 3 of the SDS gel. The crosslinked peptides comprise (a) peptide i (^72^ALGFPAPGTYAAYAR^86^) and peptide ii (^22^LLADTYTLYLK^22^) (Figure 7C), and (b) identical sequence peptides iii and iv (^22^LLADTYTLYLK^32^) (Figure 7D). Band 2 had cross-linked peptides i–ii, and band 3 included cross-linked peptides i–ii and iii–iv. The graphical representation of fragment sequences follows the commonly accepted nomenclature for peptide fragmentation [34], where peptide fragment sequences are read from left to right (*b_i_*) or from right to left (*y_i_*). Fragment sequence assignments were carried out with the aid of Proteome Discoverer (Sequest and MASCOT as search engines) by searching the MS/MS spectra against a database containing all possible Y-Y crosslinked peptides [35]. The MS/MS spectra of identified Y-Y crosslinked sequences were also searched manually with ProteinProspector, which allowed the identification of additional fragments not identified with the search engines. 

Fragment assignment is illustrated with crosslinked peptides i and ii (Figure 7C): for example, fragments *y*_5_, *y*_4_, and *y*_3_ from peptide i correspond to AAYAR, AYAR, and YAR, respectively; fragments *y*_5_, *y*_4_, and *y*_3_ from peptide ii correspond to TLYLK, LYLK, and YLK, respectively. Combining the fragments from each individual sequence allowed us to match some of the crosslinked fragments to peaks in the spectrum, such as *y*_10_ii + *y*_9_i or *y*_12_i + y_8_ii (Table S2a). A similar process led to fragment assignment of crosslinked peptides iii and iv (Figure 7D, Table S2b). These findings demonstrate the presence of Y-Y crosslinked Pa Dps subunits and implicate Y27, Y30, Y81, and Y84 as potential crosslinking residues. Mass spectrometric determination of the crosslinked residues is precluded by the presence of two Y residues in each crosslinked peptide and by the possibility that the Y-Y crosslink may be cleaved during the fragmentation reaction in the mass spectrometer.

Analysis of the Tyr network in a Pa Dps subunit dimer in the context of the Y residues implicated in Y-Y crosslinks reveals that only the pairs constituted by Y27-Y81, Y27-Y27, Y27-Y30, and Y30-Y30 are in sufficient proximity to facilitate crosslinking (Figure 7F). Visualizing these potential Y-Y crosslinks shows that these residues are within the most Tyr-dense section of the network, nearly halfway between the two ferroxidase centers (Figure 7E). Although it is probable that not all possible crosslinks are present in the sample used for the MS studies, the observations indicate that the Tyr network can function as an efficient trap for radicals formed during the oxidation of Fe^2+^ at the ferroxidase centers. 

### 2.6. Pa Dps Protects P. aeruginosa Cells from H_2_O_2_-Mediated Oxidative Stress

**Figure 8:**
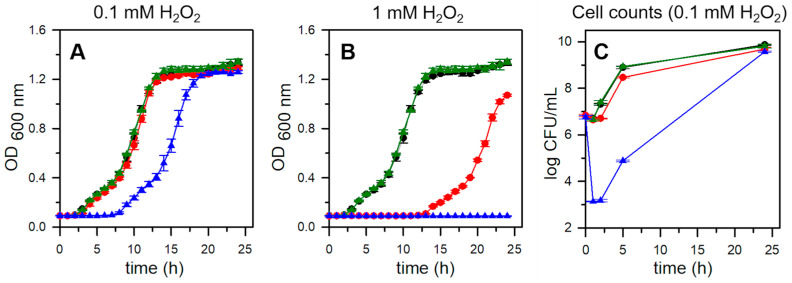
Pa Dps protects *P. aeruginosa* from H_2_O_2_-mediated oxidative stress. PAO1 (black), PAO1 + H_2_O_2_ (red), Δ*dps* (green), Δ*dps* + H_2_O_2_ (blue). Late stationary cells grown in PI media supplemented with 1 μM Fe were diluted in PI supplemented with 4 μM Fe, exposed to (**A**) 0.1 mM or (**B**) 1 mM H_2_O_2_, and cultured while monitoring the OD_600_. The extended lag phase in A and no growth in B observed with the Δ*dps* cell cultures indicate the increased sensitivity of the mutant to H_2_O_2_. Each growth curve was constructed from the average of 3 replicate wells. (**C**) Monitoring cell growth by enumerating viable cells after treatment with 0.1 mM H_2_O_2_ reveals that survival of H_2_O_2_-mediated oxidative stress is significantly aided by the presence of Pa Dps. Mean values and standard deviations are the results of three independent experiments.

To test whether Pa Dps protects *P. aeruginosa* from oxidative stress, we constructed a *P. aeruginosa* PAO1 strain with an unmarked, in-frame deletion of the PA0962 gene (Δ*dps*). The PAO1 and Δ*dps* strains were cultured to a late stationary phase (24 h) in PI media supplemented with 1 μM Fe. The cultures were diluted to OD_600_ = 0.01 in PI media and shake-incubated for 24 h. The unchallenged PAO1 and Δ*dps* cells grow at the same rate and to the same cell density (Figure 8A,B). Treatment of the PAO1 cells with 0.1 mM H_2_O_2_ does not affect growth, but a similar treatment of the Δ*dps* cells results in growth retardation (Figure 8A). When the cells are treated with 1 mM H_2_O_2_, the PAO1 cells exhibit a prolonged lag phase but eventually grow to approximately the same cell density as the untreated cells, while in contrast, the Δ*dps* cells treated with 1 mM H_2_O_2_ exhibit no growth (Figure 8B). We modified the experiments to enumerate viable cells (CFU/mL). To this end, samples were withdrawn from the cultures immediately, 1 h, 2 h, 5 h, and 24 h post-treatment, and plated on PI agar plates. The untreated PAO1 and Δ*dps* cells exhibit nearly identical CFU/mL (Figure 8C). When the cultures are treated with 0.1 mM H_2_O_2,_ the PAO1 cells show very small growth retardation, whereas the Δ*dps* exhibit a 4*log* reduction in viable cells 1 h and 2 h post-treatment. These observations indicate that the prolonged lag phase observed in Figure 8A stems from the loss of cell viability. These findings, which demonstrate that Pa Dps is important to the antioxidant stress response of *P. aeruginosa*, agree with a previous report indicating that the MvfR regulator in *P. aeruginosa* PA14, enhances protection against H_2_O_2_ by inducing the expression of antioxidant defense systems, including Dps [36].

### 2.7. Pa Dps Cleaves DNA In Vitro

It has been reported that mixing *E. coli* Dps and plasmid DNA in buffer results in large Dps-DNA complexes which do not enter 1% agarose gels [19]; these complexes are thought to be stabilized by electrostatic interactions [37]. Although the C- or N-terminal tails of some Dps proteins harbor basic residues that are thought to mediate electrostatic interactions with DNA, DNA binding by Dps is not ubiquitous because Dps from several organisms have been shown not to bind DNA [38]. The structure of Pa Dps does not include a C-terminal tail, and its N-terminal sequence does not include basic residues. The Pa Dps structure, however, has positively charged patches distributed along the Pa Dps surface where sulfate ions bind electrostatically.

**Figure 9:**
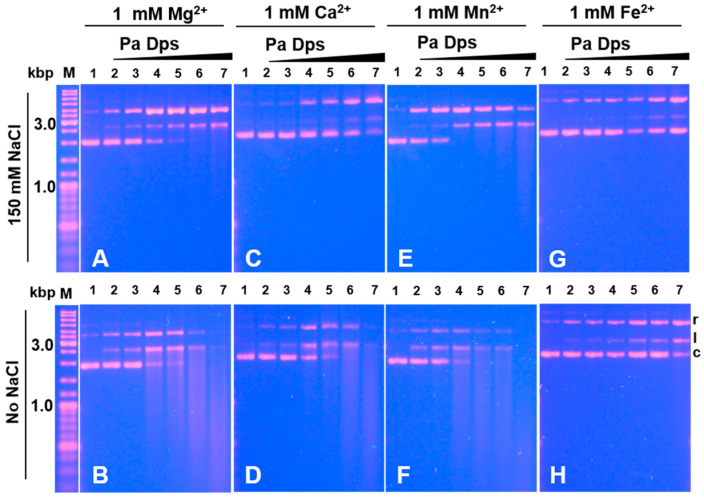
Pa Dps cleaves DNA. pUC18 plasmid DNA (8.6 nM) was incubated (1 h, 35 °C) with distinct concentrations of Pa Dps in 75 mM Tris pH 7.5 containing 150 mM NaCl and 1 mM (**A**) Mg^2+^, (**C**) Ca^2+^, (**E**) Mn^2+^, and (**G**) Fe^2+^, and in 75 mM Tris pH 7.5 containing 1mM (**B**) Mg^2+^, (**D**) Ca^2+^, (**F**) Mn^2+^, and (**H**) Fe^2+^. Samples were separated using 1% agarose gels (TBE buffer, 80 V 105 min) and stained with ethidium bromide. Lane 1 = DNA, lanes 2–7, respectively, Pa Dps:DNA mole ratio 5, 10, 50, 100, 200, and 400. Lane M = DNA electrophoresis ladder. Circular plasmid DNA is denoted by c, relaxed (nicked) DNA by r, and linear DNA by l. In experiments involving iron, Fe^2+^, Pa Dps, and DNA were incubated in an anaerobic glove box. Control experiments with bovine serum albumin (BSA) in the presence of Mg^2+^ show that the plasmid DNA remains intact (Figure S6).

To study potential interactions between Pa Dps and DNA, we incubated a constant concentration of plasmid DNA with increasing concentrations of Pa Dps in 75 mM Tris buffer (pH 7.5) containing 150 mM NaCl and 1 mM Mg^2+^. The reaction was quenched with loading dye, and the samples were separated in an agarose electrophoresis gel. These experiments revealed unexpected observations (Figure 9A): as the Pa Dps/DNA ratio increases, the intensity of the band corresponding to circular supercoiled plasmid DNA decreases and then disappears, giving rise to two new bands corresponding to relaxed (nicked) and linear DNA. When the plasmid DNA and Pa Dps are incubated in low ionic strength buffer (75 mM Tris, 1 mM MgCl_2_, pH 7.5), a similar but more pronounced degradation of the DNA is observed (Figure 9B), which is evident in the band streaking. Control experiments where plasmid DNA is incubated with bovine serum albumin (BSA) in the presence of Mg^2+^ show that the plasmid DNA remains intact (Figure S6). Similar DNA degradation occurs when Pa Dps is incubated with DNA in the presence of Ca^2+^, Mn^2+^, or Fe^2+^ at high (Figure 9C,E,G) and low (Figure 9D,F,H) ionic strength. It is also noteworthy that the anaerobic incubation of Pa Dps, DNA, and divalent metal ions (Mg^2+^, Ca^2+^, Mn^2+^, or Fe^2+^) results in gel patterns (Figure S7) nearly identical to those shown in Figure 9. These observations are significant because they indicate that DNA cleavage is likely a consequence of phosphodiester hydrolysis rather than O_2_-induced oxidative stress.

**Figure 10:**
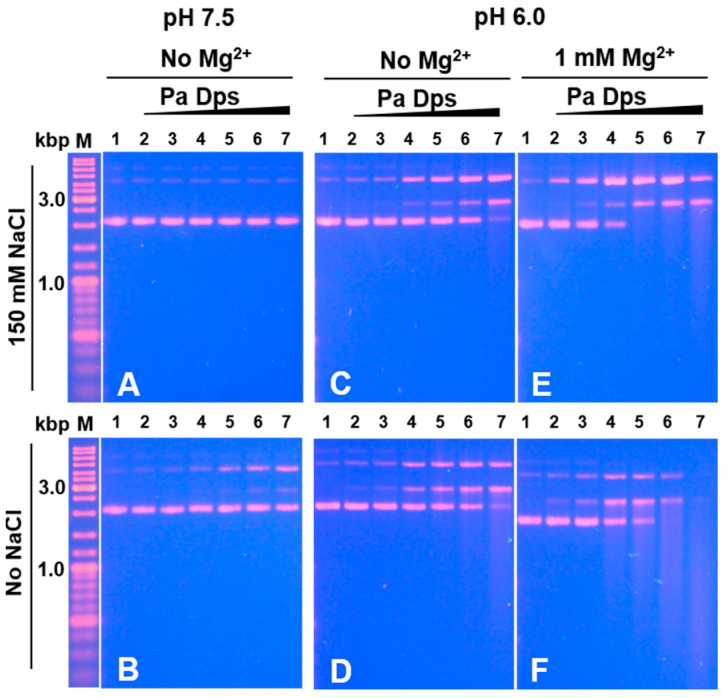
DNA cleavage requires 12-mer Pa Dps and divalent ions. Protein and pUC18 DNA plasmid (8.6 nM) were incubated in mole ratio Pa Dps:DNA = 5, 10, 50, 100, 200, and 400 (lanes 2–7, respectively) and the following conditions: (**A**) 75 mM Tris pH 7.5 and 150 mM NaCl, (**B**) 75 mM Tris pH 7.5, (**C**) 75 mM Bis-Tris pH 6.0 and 150 mM NaCl, (**D**) 75 mM Bis-Tris pH 6.0, (**E**) 75 mM Bis-Tris pH 6.0, 150 mM NaCl and 1mM MgCl_2_, and (**F**) 75 mM Bis-Tris pH 6.0 and 1 mM MgCl_2_. Lane 1 = DNA, M = DNA ladder.

To understand whether 12-mer Pa Dps assembly is required for DNA cleaving activity, we incubated DNA and Pa Dps in the absence of divalent metal ions at pH 7.5 and at pH 6.0, capitalizing from our findings indicating that divalent metal ions are not required for 12-mer assembly at pH 6.0 but are required at pH 7.5 (see Figure 1). Experiments in the absence of divalent metal ions at pH 7.5 and low ionic strength show the emergence of nicked DNA when the Pa Dps/DNA mole ratio is ~100 (Figure 10B), whereas, at high ionic strength, the plasmid DNA is not affected (Figure 10A). Comparing these results to those obtained at pH 7.5 in the presence of Mg^2+^ (Figure 9A,B) reveals that 12-mer Pa Dps exhibits significantly higher DNA-cleaving activity. Experiments at pH 6.0 in the absence of divalent cations (Figure 10C,D) show higher DNA cleaving activity than that observed with disassembled Pa Dps at pH 7.5 (Figure 10A,B) but lower than that observed with 12-mer Pa Dps in the presence of Mg^2+^ (Figure 9). Consequently, we incubated Pa Dps and DNA at pH 6.0 in the presence of Mg^2+^ (Figure 10E,F) and observed a DNA cleaving pattern similar to that seen at pH 7.5 in the presence of Mg^2+^. 

Taken together, these observations indicate that the DNA-cleaving activity of Pa Dps is enhanced by the 12-mer formation and by the presence of divalent ions. Although understanding the reaction mechanism will require additional work, it is tempting to speculate that divalent cations exert their influence by mediating the DNA-Pa Dps interactions and probably facilitating the hydrolysis of phosphodiester bonds. In this context, it is interesting to note that Fe^2+^ is less effective than Mg^2+^, Ca^2+^, and Mn^2+^ at facilitating DNA cleavage, observations that are consistent with the preference of DNA to bind Mg^2+^, Ca^2+^, and Mn^2+^ ions [39].

### 2.8. Pa Dps Does Not Contribute to the Utilization of DNA as a Nutrient Source

**Figure 11:**
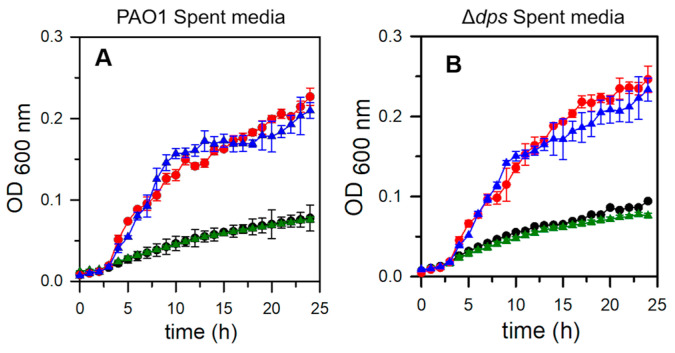
Pa Dps does not contribute to the utilization of DNA as a nutrient source. PAO1 (black), PAO1 + DNA (red), Δ*dps* (green), Δ*dps* + DNA (blue). The growth of PAO1 or Δ*dps* cells in spent media obtained from culturing PAO1 (**A**) or Δ*dps* (**B**) cells is aided similarly by the presence of DNA as a nutrient source. Each of the growth curves was constructed from the average and standard deviation of 3 replicate wells.

The DNA-binding assays described above showed that instead of forming a stable DNA-Dps complex, Pa Dps could cleave the DNA in vitro. This novel observation led us to ask whether Pa Dps may function to facilitate the utilization of DNA as a nutrient source when the bacteria face starvation conditions. Consequently, we studied the ability of PAO1 and Δ*dps* strains to use exogenous DNA added to starved cell cultures. To ensure that bacteria were starved, we first cultured the cells to a late stationary phase (44 h) in PI broth supplemented with 1 μM Fe, a condition that has been demonstrated triggers starvation [14,15]. The cultures were centrifuged, the starved cells were separated from the supernatants (spent media), and the spent media was filter sterilized. The sterile spent media was subsequently used to culture the starved cells in the presence and absence of salmon sperm DNA (Figure 11). Utilizing spent media ensures that proteins that may have been secreted either by normal secretion pathways or by lysis of starved cells are present in the growth media. The results show that the addition of 1 mg mL^−1^ DNA to spent media increases the growth of both PAO1 and Δ*dps* cells similarly, but there is no difference in the growth of PAO1 and mutant cells. These observations suggest that the deletion of *dps* did not affect the ability of the bacteria to use DNA as a nutrient source, so it is probable that Dps in *P. aeruginosa* is not required to degrade extracellular DNA and liberate accessible sources of phosphate, carbon, and nitrogen to promote growth.

## 3. Materials and Methods

### 3.1. Chemicals, Bacterial Strains, and Media

Chemicals were purchased from Fisher Scientific (Waltman, MA, USA) unless otherwise indicated. *Pseudomonas aeruginosa* PAO1-UW25 [40] was purchased from the University of Washington Genome Center. A PAO1-derived strain with an unmarked, in-frame deletion of the *dps* gene (PA0962) was made using methods described previously [41]. Briefly, the plasmid pEXG2 [42] was used to deliver a synthetic DNA fragment (Genscript) flanking the PA0962 gene to the *P. aeruginosa* chromosome. The pEXG2 was transformed into *E. coli* S17-1 (λpir) and then crossed into PAO1 by mating. Transconjugants were selected on PI agar containing gentamicin (100 μg/mL), and deletion mutants were selected using no-salt LB agar containing 10% (wt/vol) sucrose. All *P. aeruginosa* strains were kept on Pseudomonas Isolation Agar (PIA) (BD Biosciences), and Pseudomonas Isolation (PI) media (20 g L^−1^ peptone, 0.3 g L^−1^ MgCl_2_·6H_2_O, 10 g L^−1^ K_2_SO_4_, 25 mg L^−1^ irgasan, and 20 mL L^−1^ glycerol, pH 7.0) was used for normal growth conditions. When required, iron supplementation was carried out by addition of a small volume of a filter-sterilized solution of 10 mM (NH_4_)_2_Fe(SO_4_)_2_ (pH∼2.0). 

The gene encoding Pa Dps (PA0962) was synthesized, subcloned into a pET11a vector, and sequenced (GeneScript Corp., Piscataway, NJ, USA). The gene was engineered with silent mutations introducing codons favored by *E. coli* [43] and with NdeI and BamHI restriction sites at the 5′ and 3′ ends, respectively. The pET11a-*dps* construct was transformed into *E. coli* BL21DE3 Gold cells (Agilent Technologies) for protein expression.

### 3.2. Expression and Purification of Pa Dps

Pre-cultures were grown overnight (37 °C, 220 rpm) from a single colony of *E. coli* BL21DE3 Gold cells transformed with the pET11a-*dps* construct in 50 mL of LB medium containing 50 μg/mL ampicillin. The 50 mL pre-cultures were used to inoculate 1 L of fresh LB media. The expression cultures (200 rpm, 37 °C) were induced with 0.5 mM isopropyl β-D-1-thiogalactopyranoside (IPTG) when the optical density at 600 nm (OD_600_) reached 0.6–0.8. After 4 h, the cells were harvested by centrifugation and stored at −20 °C. Cell paste was resuspended in lysis buffer (50 mM Tris-HCl, 150 mM NaCl, 1 mM EDTA, 1% Triton X-100, 1.6 mg/g cell paste lysozyme, 4 mg/g cell paste deoxycholic acid, 0.5 mM DTT, and protease inhibitor tablet, pH 7.5) and lysed by ultrasound in an ice bath with the aid of a Qsonica Q500 sonicator operating with a 60% pulse amplitude and 10 cycles of pulse-on (15 s) and pulse-off (45 s). The cell lysate was clarified by centrifugation (63,000× *g*) for 45 min at 4 °C, and the supernatant was subjected to a 40% (NH_4_)_2_SO_4_ cut (40 min) in an ice bath. The resultant sample was centrifuged (64,000× *g*) for 40 min, and the precipitate was resuspended in buffer A (20 mM Tris-HCl containing 50 mM NaCl, pH 7.5) and dialyzed against the same buffer. The resultant solution was filtered through a 4 μm nylon filter (VWR) prior to loading onto a 10 mL HiTrap™ Q-Sepharose column (GE Healthcare) equilibrated with buffer A. The column was then washed with buffer A (5 column volumes) and eluted with buffer A and a gradient of 50–400 mM NaCl (20 column volumes). Elution fractions containing Pa Dps were dialyzed against buffer A and processed a second time through a 10 mL HiTrap™ Q-Sepharose column using the above-described conditions. Eluant fractions containing Dps were purified to homogeneity in a size exclusion column (Superdex 200 Increase 10/300 GL), equilibrated, and eluted with buffer B (75 mM Tris, 150 mM NaCl, pH 7.0 at 4 °C). Pa Dps fractions eluting from this column contain pure protein as ascertained by a single band in SDS PAGE experiments. Protein concentration was determined using a Pierce™ BCA Protein Assay Kit (Thermo Scientific, Waltham, MA, USA). Pure protein stock was aliquoted in Eppendorf tubes, flash-frozen in liquid nitrogen, and stored at −80 °C.

### 3.3. Titration of Pa Dps with Fe^2+^ under Aerobic and Anaerobic Conditions

Anaerobic titrations were carried out in an anaerobic glove box (Coy): 12-mer Pa Dps (2.0 mL, 2.0 μM) in 75 mM Tris (pH 7.5) containing 1 mM MgCl_2_ was placed in a 1 cm cuvette containing a magnetic bar. The solution was titrated with FeCl_2_ (20 mM) and H_2_O_2_ (10 mM). Each aliquot delivered 50 Fe^2+^/12-mer followed by 1 equivalent of H_2_O_2_ relative to Fe^2+^. The solution was stirred for 2 min after the addition of each aliquot prior to recording UV-vis spectra with the aid of a Cary 50 spectrophotometer. The resultant solution was desalted through a Sephadex G25 M column and concentrated to 2.0 mL. An amount of 50 μL was utilized to determine the iron content using a Ferrozine assay [27], and 20 μL was used to determine the protein concentration. The remaining solution was chromatographed through a Superdex S200 column equilibrated and eluted with 75 mM Tris, 1 mM MgCl_2_, pH 7.5 at 4 °C). Protein eluting from the column was separated in an SDS PAGE gel and stained with Coomassie brilliant blue. Select bands were excised from the gel and used for experiments described in Section 3.4. Aerobic titrations were carried out similarly, except that dissolved O_2_, instead of H_2_O_2_, acted as the oxidant. 

### 3.4. Proteomics Analysis of Di-Tyrosine Crosslinked Pa Dps

Protein in the excised bands was reduced with DTT, alkylated with iodoacetamide (IAA, Sigma), and digested overnight with sequencing grade trypsin/lysozyme-C enzyme combo (Promega) [44]. After acidification with 5% formic acid in acetonitrile (1:2 vol/vol), samples were dried under vacuum and stored at −80 °C.

Dried extracts were dissolved in 10 µL 0.1% formic acid; 5 µL were used for LC-MS/MS analysis on a Thermo Scientific Q-Exactive orbitrap mass spectrometer coupled to an Ultimate 3000 RSLC liquid chromatography. The mobile phase composition: A = 0.1% formic acid in water, B = 0.1% formic acid in acetonitrile. The peptides were loaded onto an Acclaim PepMap 100 C18 trap cartridge (0.3 × 5 mm, 5 µm particle size, 100 Å pore size) and transferred using 2% B (5 min) at 20 µL/min. Separation was carried on an Acclaim PepMap 100 C18 nanoLC column (0.075 × 25 mm, 3 µm particle size, 100 Å pore size) using a linear gradient (5% to 45% B, 30 min) at 300 nL/min. The mass spectrometer was operated in data-dependent acquisition with a loop count of 10. Full MS parameters: resolution = 70,000, ACG = 3 × 10^5^, scan range = 375–1600 *m*/*z*, maximum injection time = 50 ms; data-dependent acquisition parameters: resolution = 17,500, AGC target = 1 × 10^5^, isolation window = 1.6 *m*/*z*, maximum injection time = 110 ms, normalized collision energy = 28, dynamic exclusion = 60 s, peptide match = preferred, exclude isotopes = on, charge exclusion = unassigned 7, 8, >8, and maximum AGC target = 1.1 × 10^3^, which resulted in an intensity threshold of 1.0 × 10^4^.

Data analysis was conducted in Proteome Discoverer (ver. 2.4, Thermo Scientific) with Sequest HT and Mascot (Matrix Science, London, UK). Data were searched against a custom database generated using X-Comb [35], as previously described [45]: the protein sequence (Uniprot ID Q9I4Z7, Pseudomonas Genome ID PA0962) was used to generate a database with all possible crosslinked peptides, selecting the following options: trypsin, maximum of 2 missed cleavages, inter- and intra-crosslinking, and a minimum of 5 amino acids/peptide. The resulting FASTA file contains the sequences of crosslinked peptides as if linked by a peptide bond rather than a tyrosine (Y-Y) crosslink. Given that formation of a peptide bond results in the loss of a water molecule (18.0106 Da), whereas a Y-Y crosslink results only in the loss of 2 H atoms (2.0156 Da), we adopted the same variable modification of Y+7.9975 Da described by Mukherjee et al. [45] to account for the difference in mass between the database of crosslinked sequences and the MS data. The database of crosslinked peptides was searched, allowing for Met oxidation (dynamic modification) and cysteine carbamidomethylation (static modification). Sequest HT parameters: minimum peptide length = 5, precursor mass tolerance = 20 ppm, fragment mass tolerance = 0.2 Da, use average precursor and fragment mass = false, spectrum matching ions = NL A ion–NL B ions–NL y ions–flanking ions. Mascot parameters: precursor mass tolerance = 20 ppm, fragment mass tolerance = 0.2 Da; use average precursor and fragment mass = false. Percolator was used to perform target/decoy analysis and calculate false discovery rates (FDR), which were used to generate two levels of target FDR: strict = 0.01 and relaxed = 0.05. Percolator parameters: Target/Decoy selection = concatenated, Validation based on = q-Value, maximum Delta Cn = 0.05, maximum rank = 0.

MS/MS spectra identified as potential matches by Proteome Discoverer were further searched using the MS-Product algorithm of the ProteinProspector suite (http://prospector.ucsf.edu, accessed on 20 January 2023). The parameters were set as those described for Sequest and MASCOT. The charge was specified depending on the peptide under investigation. This step was employed to assign fragments coming from each individual sequence comprising the crosslinked peptide. Therefore, each sequence was indicated separately.

### 3.5. Crystallization and Data Collection

Crystallization screening was carried out at 18 °C with purified Dps, 5 mg/mL in (i) 75 mM Tris, 200 mM NaCl, and pH 7.5 or (ii) 75 mM Tris, 0.5 mM MnCl_2_, and pH 7.5, using an NT8 drop setting robot (Formulatrix, Inc., Bedford, MA, USA) and UVXPO MRC (Molecular Dimensions) and sitting drop vapor dimensions plates. Protein and crystallization solution (100 nL each) were dispensed and equilibrated against 50 µL of the latter.

Crystals were obtained from Dps in 75 mM Tris, 200 mM NaCl, pH 7.5. This solution produced the following crystal: (a) prismatic crystals of a primitive orthorhombic form (Dps-o); (b) primitive cubic lattice crystals (Dps-c); (c) primitive cubic crystals were used to prepare Dps-iron complexes (Dps-c-Fe); (d) Dps-o-Fe was prepared from Dps-o crystals. 

Dps-o crystals grew in approximately 1 week from the Proplex HT screen (Molecular Dimensions) condition G5 (1 M (NH_4_)_2_SO_4_, 1M KCl, 100 mM HEPES pH 7.0). The crystals were transferred to a cryoprotectant solution composed of 4 M (NH_4_)_2_SO_4_ before being stored in liquid nitrogen. Dps-c crystals were obtained from the Index HT screen (Hampton Research) condition C9 (1.1 M sodium malonate, 100 mM Hepes pH 7.0, 0.5% (*v*/*v*) Jeffamine ED-2001). The crystals were vitrified in a cryoprotectant solution composed of 80% (*v*/*v*) crystallant and 20% (*v*/*v*) PEG 200. Crystals of iron-Dps complexes Dps-o-Fe and Dps-c-Fe were prepared, respectively, from Dps-o crystals noted above and from Dps primitive cubic crystals obtained from the Proplex HT (Molecular Dimensions) screen condition E4 (8% (*w/v*) PEG 8000, 100 mM Hepes pH 7.0). To this end, crystals were transferred to a solution containing 80% crystallant, 20% PEG 200, and 50 mM FeCl_2_ for Dps-c-Fe and 80% crystallant, 20% glycerol, and 50 mM FeCl_2_ for Dps-o-Fe. The crystals were soaked for 10 min in this solution before harvesting and storing in liquid nitrogen. 

Crystals were obtained from Dps in 75 mM Tris, 0.5 mM MnCl_2_, and pH 7.5. This solution produced prismatic crystals of cubic lattice (Dps-c-Mn) in approximately 1 week from the Index HT screen (Hampton Research) condition C7 (0.8 M Na/K tartrate, 100 mM Tris pH 8.5, 0.5% (*w/v*) PEG 5000 MME). Samples were transferred to a cryoprotectant solution composed of 80% (*v*/*v*) crystallant and 20% (*v*/*v*) glycerol.

X-ray diffraction. Data were collected at the Advanced Photon Source IMCA-CAT beamline 17-ID using an Eiger2 X 9M pixel array detector. Data for PA0962-c-Fe were collected at wavelengths of 1.0000 Å for refinement, and 1.5498 Å for calculation of anomalous difference maps to locate the iron atom positions. Although this wavelength is on the high-energy side of the iron X-ray fluorescence spectrum, there is an appreciable signal (3.185 e^−^). Diffraction data for Dps-o-Fe and Dps-c-Mn were collected at the National Synchrotron Light Source-II (NSLS-II) NYX beamline 19-ID using an Eiger2 XE 9M pixel array detector.

### 3.6. Structure Solution and Refinement

Intensities were integrated using XDS [46,47], and the Laue class analysis and data scaling were conducted with Aimless [48]. The structure solution for Dps-c was conducted by molecular replacement with Phaser [49] using a single subunit of a previously determined DPS protein from *Bacillus brevis* (PDB 1N1Q) as the search model. The top solution was obtained in the space group *P*2_1_3 and contained twelve molecules (dodecamer) plus a third of a dodecamer (4 molecules) in the asymmetric unit for a total of 16 molecules. The Dps-o structure was solved by molecular replacement using a single subunit of the Dps-c structure as a search model. The top solution was obtained in the space group *P*2_1_2_1_2_1_ with twelve molecules in the asymmetric unit. The models were improved by automated model building with Phenix [50], and final structures were obtained by additional refinement and manual model building with Phenix and Coot [51], respectively. Occupancies were refined for the iron atoms in the Dps-c-Fe structure. Disordered side chains were truncated to the point for which electron density could be observed. Structure validation was conducted with Molprobity [52], and figures were prepared using the CCP4MG package [53]. Structure superposition was conducted with GESAMT [54]. Crystallographic data are provided in Table S1.

### 3.7. Tolerance of P. aeruginosa PAO1 and Δdps Strains to H_2_O_2_

Starter cultures (5 mL) of PAO1 and ∆*dps* cells were grown overnight from a single colony (37 °C, 220 rpm) in PI media supplemented with 10 μM Fe. The overnight cultures were diluted to OD_600_ = 0.001 and then cultured (5 mL) for 24 h in PI media supplemented with 1 μM Fe at the same temperature and shaking speed. The 24 h cultures were used as follows: (i) Diluted to OD_600_ = 0.01 in 5 mL PI media supplemented with 4 μM Fe and treated with a 100 mM H_2_O_2_ solution to render 0.1 mM H_2_O_2_ final concentration. Samples (100 μL) from each culture were collected before (*t* = 0) and after H_2_O_2_ addition (*t* = 1 h, 2 h, 5 h, 24 h), diluted, and plated on PIA to enumerate viable cells. Colony forming units (CFUs) after 12 h incubation at 37 °C were used to calculate the percentage of survival. (ii) Diluted to OD_600_ = 0.01 in PI media supplemented with 4 μM Fe and treated with a 10 mM or 100 mM H_2_O_2_ solution to render 0.1 mM or 1 mM H_2_O_2_ final concentrations, respectively. The resultant cultures were placed in a 96-well plate (200 μL per well, triplicate wells for each condition), and the plate was inserted in a BioTek EPOCH2 microplate reader for culturing at 37 °C and 205 cpm, recording OD_600_ every hour.

### 3.8. Utilization of DNA as a Nutrient Source

A single colony of PAO1 or ∆*dps* cells was used to inoculate 5 mL of PI broth supplemented with 1 μM Fe in a 50 mL conical tube sealed with an oxygen-permeable membrane, and the cells were cultured for 42 h (37 °C, 220 rpm). The cultures were centrifuged (3148× *g*, 4 °C, 15 min), and the clarified spent media was sterilized by filtration (Nalgene^®^ Syringe Filters, Nylon, 0.2 μm, Thermo Scientific). The cells were resuspended in phosphate buffer saline (PBS pH 7.4), centrifuged (3148× *g*, 4 °C, 15 min), resuspended in 5 mL PBS and the OD_600_ adjusted to 1 with the same buffer. The resultant cell suspension was diluted 100 times with the filter-sterilized spent media to OD_600_ = 0.01 containing 1 mg mL^−1^ of ultrapure salmon sperm DNA (Invitrogen™) and transferred to 96-well plates (200 μL per well, triplicate wells per condition). The microwell plate was placed in a BioTek EPOCH2 microplate reader to culture the cells (37 °C 205 cpm), recording the OD_600_ every hour.

## 4. Conclusions

Our findings demonstrate that gene PA0962 in *P. aeruginosa* PAO1 encodes a protein that exhibits the characteristic Dps fold. At neutral pH and in the presence of divalent metal ions, the protein adopts the distinctive 12-mer quaternary structure (Pa Dps) that harbors two di-iron ferroxidase sites at the interface of each subunit dimer, with iron ligands provided by residues in each of the subunits and with Fe1 exhibiting higher average refined occupancy than Fe2. The site occupied by Fe1 in the di-iron centers also accepts Na^+^ or Mn^2+^ ions, which are coordinated by the same ligands H49 from one subunit and D64 and E68 from the accompanying subunit. The oxidation of iron is catalyzed by the ferroxidase centers when the oxidant is H_2_O_2_ (not O_2_). The higher reactivity of Pa Dps ferroxidase centers toward H_2_O_2_ relative to O_2_ has also been observed with other Dps molecules [55,56]. The preference for peroxide is thought to be related to the ferroxidase center structure, but the details are not yet understood. Although the reaction catalyzed by the ferroxidase centers can be expected to significantly decrease the formation of hydroxyl radicals, these are not completely suppressed. The latter is made evident by the crosslinking of tyrosine residues that occurs when Pa Dps, Fe^2+^, and H_2_O_2_ are reacted. In this context, the Pa Dps structure shows a network of Tyr residues contributed by both subunits of a subunit dimer and in proximity to both ferroxidase centers in the subunit dimer. This network of Tyr residues functions to efficiently “soak” radicals that may be generated when Fe^2+^ is oxidized at the Pa Dps ferroxidase center. Together, the ferroxidase centers and the network of Tyr residues are expected to endow Pa Dps with the ability to fend off H_2_O_2_-mediated oxidative stress, a prediction that has been fulfilled in experiments demonstrating that the Δ*dps* mutant strain of *P. aeruginosa* is significantly more susceptible to H_2_O_2_ stress than the wild-type counterpart. Finally, a unique observation is the exonuclease activity observed when 12-mer Pa Dps and DNA are incubated in vitro. The contribution of this activity to the *P. aeruginosa* cell is not yet evident, but we speculate that the DNAse activity of Pa Dps may serve the cell in the biofilm lifestyle when nutrients become severely limiting and cells leave the biofilm matrix formed by extracellular DNA, proteins, and polysaccharides.

## Data Availability

Coordinate and structures factors for the following Pa Dps structures were deposited to the Worldwide Protein Databank with accession codes Dps-o (8FF9), Dps-c (8FFA), Dps-o-Fe (8FFB), Dps-c-Fe (8FFC) and Dps-c-Mn (8FFD).

## Supplementary Materials

The supporting information can be downloaded at: https://www.mdpi.com/article/10.3390/ijms24054669/s1. References [57,58,59,60,61] are cited in the supplementary materials.
