# Supplementary material for: Pseudomonas aeruginosa Dps (PA0962) Functions in H2O2 Mediated Oxidative Stress Defense and Exhibits In Vitro DNA Cleaving Activity
Source: Int J Mol Sci. 2023 Feb 28;24(5):4669. doi: 10.3390/ijms24054669 (PMC10002758; doi:10.3390/ijms24054669)
Supplement: Supplementary file 1 [file ijms-24-04669-s001.zip › ijms-2223496-supplementary.pdf]

# Supplementary Information

# *Pseudomonas aeruginosa* Dps (PA0962) functions in $H_2O_2$ mediated oxidative stress defense and exhibits *in vitro* DNA cleaving activity.

***Nimesha Rajapaksha [1], Anabel Soldano [1], Huili Yao [1], Fabrizio Donnarumma [1], Maithri M. Kashipathy [2], Steve Seibold [2], Kevin P. Battaile [3], Scott Lovell [2], and Mario Rivera [1,*]***

[1] Department of Chemistry, Louisiana State University, 232 Choppin Hall, Baton Rouge, LA, 70803, USA

[2] Protein Structure and X-ray Crystallography Laboratory, University of Kansas, 2034 Becker Dr., Lawrence, KS 66047

[3] NYX, New York Structural Biology Center, Upton, NY, 11973, USA

*Corresponding author E-mail: mrivera@lsu.edu

ORCID: 0000-0002-5692-5497

**Table S1**. Crystallographic data for Pa Dps structures.

| **Structure** | **Dps-o** | **Dps-c** | **Dps-o-Fe** | **Dps-c-Fe** | **Dps-c-Mn** |
|---|---|---|---|---|---|
| **PDB Code** | **8FF9** | **8FFA** | **8FFB** | **8FFC** | **8FFD** |
| **Data Collection** | | | | | |
| Unit-cell parameters (Å, °) | *a*=129.54 *b*=129.82 *c*=156.60 | *a*=*b*=*c*=223.46 | *a*=131.08 *b*=131.84 *c*=157.82 | *a*=*b*=*c*=223.90 | *a*=*b*=*c*=223.91 |
| Space group | $P2_12_12_1$ | $P2_13$ | $P2_12_12_1$ | $P2_13$ | $P2_13$ |
| Resolution (Å)[1] | 48.43-1.70 (1.73-1.70) | 49.97-2.15 (2.19-2.15) | 48.86-2.25 (2.29-2.25) | 48.86-1.85 (1.88-1.85) | 48.86-2.20 (2.24-2.20) |
| Wavelength (Å) | 1.0000 | 1.0000 | 0.9795 | 1.0000 | 0.9786 |
| Temperature (K) | 100 | 100 | 100 | 100 | 100 |
| Observed reflections | 1,964,310 | 4,163,876 | 866,553 | 6,542,757 | 1,964,631 |
| Unique reflections | 288,462 | 200,207 | 129,943 | 315,151 | 188,113 |
| $\langle I/\sigma(I)\rangle$[1] | 10.8 (1.8) | 12.5 (1.7) | 11.2 (1.6) | 15.2 (1.9) | 10.2 (1.9) |
| Completeness (%)[1] | 100 (100) | 100 (100) | 100 (100) | 100 (100) | 100 (100) |
| Multiplicity[1] | 6.5 (6.9) | 20.8 (21.4) | 6.7 (6.5) | 20.8 (21.0) | 10.4 (9.5) |
| $R_{merge}$ (%)[1, 2] | 10.5 (115.9) | 20.2 (234.3) | 12.8 (124.5) | 14.9 (198.5) | 18.8 (127.2) |
| $R_{meas}$ (%)[1, 4] | 11.4 (125.2) | 20.7 (240.0) | 13.9 (135.3) | 15.3 (203.5) | 19.8 (134.6) |
| $R_{pim}$ (%)[1, 4] | 4.3 (46.9) | 4.5 (51.8) | 5.3 (52.6) | 3.4 (44.4) | 6.1 (43.6) |
| $CC_{1/2}$[1, 5] | 0.997 (0.632) | 0.999 (0.612) | 0.997 (0.625) | 0.999 (0.684) | 0.997 (0.638) |
| **Refinement** | | | | | |
| Resolution (Å)[1] | 48.43-1.70 | 49.97-2.13 | 48.86-2.25 | 43.91-1.85 | 48.86-2.20 |
| Reflections (working/test)[1] | 273,890/14,417 | 190,062/10,047 | 123,391/6,434 | 299,344/15,615 | 178,615/9,437 |
| $R_{factor}$ / $R_{free}$ (%)[1,3] | 14.7/17.0 | 18.3/22.7 | 18.3/23.3 | 16.2/19.2 | 17.0/22.4 |
| No. of atoms (Protein/Sulfate/Iron/Water) | 14,667/405/-/2,323 | 19,175/-/-/1,163 | 14,366/-/24/765 | 19,515/-/63/2,111 | 19,504/16/2,101 |
| **Model Quality** | | | | | |
| R.m.s deviations | | | | | |
| Bond lengths (Å) | 0.009 | 0.011 | 0.009 | 0.010 | 0.009 |
| Bond angles (°) | 0.946 | 1.050 | 0.917 | 0.989 | 0.962 |
| Average *B*-factor (Å$^2$) | | | | | |
| All Atoms | 24.6 | 41.9 | 39.6 | 29.3 | 33.4 |
| Protein | 22.3 | 41.8 | 39.6 | 28.4 | 32.8 |
| Sulfate | 55.0 | - | - | - | 43.8 |
| Iron | - | - | 60.6 | 50.0 | 38.7 |
| Water | 34.3 | 44.6 | 39.7 | 37.3 | 0.27 |
| Coordinate error(maximum likelihood) (Å) | 0.16 | 0.26 | 0.31 | 0.18 | |
| Ramachandran Plot | | | | | 97.2 |
| Most favored (%) | 98.3 | 97.0 | 98.0 | 97.6 | 2.6 |
| Additionally allowed (%) | 1.7 | 2.9 | 2.0 | 2.4 | |

1) Values in parenthesis are for the highest resolution shell.

2) $R_{merge} = \Sigma_{hkl}\Sigma_i |I_i(hkl) - \langle I(hkl)\rangle| / \Sigma_{hkl}\Sigma_i I_i(hkl)$, where $I_i(hkl)$ is the intensity measured for the *i*th reflection and $\langle I(hkl)\rangle$ is the average intensity of all reflections with indices hkl.

3) $R_{factor} = \Sigma_{hkl} ||F_{obs}(hkl)| - |F_{calc}(hkl)|| / \Sigma_{hkl} |F_{obs}(hkl)|$; Rfree is calculated in an identical manner using 5% of randomly selected reflections that were not included in the refinement.

4) $R_{meas}$ = redundancy-independent (multiplicity-weighted) $R_{merge}$[1, 2]. $R_{pim}$ = precision-indicating (multiplicity-weighted) $R_{merge}$[3, 4].

5) $CC_{1/2}$ is the correlation coefficient of the mean intensities between two random half-sets of data [5, 6].

## References


1. Evans, P.R., *An introduction to data reduction: space-group determination, scaling and intensity statistics.* Acta Crystallogr D Biol Crystallogr, 2011. **67**(Pt 4): p. 282-92.
2. Evans, P., *Scaling and assessment of data quality.* Acta Crystallogr D Biol Crystallogr, 2006. **62**(Pt 1): p. 72-82.

3. Diederichs, K. and P.A. Karplus, *Improved R-factors for diffraction data analysis in macromolecular crystallography*. Nat Struct Biol, 1997. **4**(4): p. 269-75.
4. Weiss, M.S., *Global indicators of X-ray data quality*. Journal of Applied Crystallography, 2001. **34**: p. 130-135.
5. Karplus, P.A. and K. Diederichs, *Linking crystallographic model and data quality*. Science, 2012. **336**(6084): p. 1030-3.
6. Evans, P., *Biochemistry. Resolving some old problems in protein crystallography*. Science, 2012. **336**(6084): p. 986-7.

**Table S2a**

Fragments from tandem mass spectra of crosslinked peptide i-ii

| Fragment type | Fragment sequence | Mass (Da) | Charge | Intensity (A.U.) |
|---|---|---|---|---|
| **A L G F P A P G T Y A A Y A R** | | | | |
| RA | $y_2$ | 246.1552 | +1 | 18474 |
| RAY | $y_3$ | 409.2198 | +1 | 32587 |
| RAYA | $y_4$ | 480.257 | +1 | 34361 |
| RAYAA | $y_5$ | 551.2946 | +1 | 43716 |
| RAYAAYTGPAPFG | $y_{13}$ | 671.3396 | +2 | 2793 |
| ALG | $b_3$ | 242.15 | +1 | 108750 |
| ALGF | $b_4$ | 389.2184 | +1 | 11428 |
| ALGFP | $b_5$ | 486.2727 | +1 | 2363 |
| **L L A D T Y T L Y L K** | | | | |
| KL | $y_2$ | 260.1979 | +1 | 28211 |
| KLY | $y_3$ | 423.2595 | +1 | 34934 |
| KLYL | $y_4$ | 536.3452 | +1 | 15069 |
| KLYLT | $y_5$ | 637.3922 | +1 | 13858 |
| LL | $b_2$ | 227.1756 | +1 | 48856 |
| LLA | $b_3$ | 298.2131 | +1 | 5526 |
| LLAD | $b_4$ | 413.2427 | +1 | 5793 |
| LLADT | $b_5$ | 514.2853 | +1 | 3527 |
| LLADTYTLY | $b_9$ | 1054.539 | +1 | 5432 |
| **LLADTYTLYLKPGTYAAYAR** | $y_{10}$ii + $y_9$i | 723.0387 | +3 | 14370 |
| **FPAPGTYAAYARDTYTLYLK** | $y_{12}$i + $y_8$ii | 766.3679 | +3 | 2899 |
| **FPAPGTYAAYARLK** | $y_{12}$i + $y_2$ii | 771.862 | +2 | 5261 |
| **ALGFPAPGTYAAYARYTLYLK** | i +$y_6$ii | 775.381 | +3 | 4557 |
| **ALGFPAPGTYAAYARTYTLYLK** | i +$y_7$ii | 808.8776 | +3 | 3473 |
| **LADTYTLYLKAYAR** | $y_{10}$ii + $y_4$i | 839.8978 | +2 | 3917 |
| **FPAPGTYAAYARYLK** | $y_{12}$i + $y_3$ii | 852.4079 | +2 | 5402 |
| **ALGFPAPGTYAAYARLK** | i +$y_2$ii | 891.9276 | +2 | 4409 |
| **LLADTYTLYLKYAAYAR** | ii + $y_6$i | 1013.544 | +2 | 12093 |
| **LLADTYTLYLKTYAAYAR** | ii + $y_7$i | 1063.551 | +2 | 13512 |
| **LADTYTLYLKPAPGTYAAYAR** | $y_{10}$ii + $y_{11}$i | 1168.594 | +2 | 9231 |
| **LADTYTLYLKPGTYAAYAR** | $y_{10}$ii + $y_9$i | 1084.564 | +2 | 21901 |
| **FPAPGTYAAYARTYTLYLK** | $y_{12}$i + $y_7$ii | 1092.575 | +2 | 5109 |
| **LLADTYTLYLKPGTYAAYAR** | ii + $y_9$i | 1140.6 | +2 | 74957 |
| **LLADTYTLYLKFPGTYAAYAR** | ii + $y_{10}$i | 1176.134 | +2 | 11356 |

**Table S2b**

Fragments from tandem mass spectra of crosslinked peptide iii-iv

| Fragment type | Fragment sequence | Mass (Da) | Charge | Intensity (A.U.) |
|---|---|---|---|---|
| **L L A D T Y T L Y L K** | | | | |
| KL | $y_2$ | 260.1969 | +1 | 35056 |
| KLY | $y_3$ | 423.2598 | +1 | 32766 |
| KLYL | $y_4$ | 536.3437 | +1 | 10678 |
| KLYLT | $y_5$ | 637.3944 | +1 | 10633 |
| KLYLTY | $y_6$ | 800.4426 | +1 | 4050 |
| KLYLTYTD | $y_8$ | 1016.531 | +1 | 6824 |
| KLYLTYTDA | $y_9$ | 1087.565 | +1 | 42533 |
| KLYLTYTDAL | $y_{10}$ | 1200.641 | +1 | 5792 |
| LL | $b_2$ | 227.1756 | +1 | 60687 |
| LLA | $b_3$ | 298.2123 | +1 | 3546 |
| LLAD | $b_4$ | 413.241 | +1 | 3441 |
| **LADTYTLYLKYLK** | $y_{10}$iii + $y_3$iv | 810.374 | +2 | 3531 |
| **ADTYTLYLKLYLK** | $y_9$iii + $y_4$iv | 810.9352 | +2 | 7966 |
| **ADTYTLYLKTLYLK** | $y_9$iii + $y_5$iv | 861.4788 | +2 | 2801 |
| **LLADTYTLYLKLYLK** | iii +$y_4$iv | 924.0405 | +2 | 2793 |
| **ADTYTLYLKYTLYLK** | $y_9$iii + $y_6$iv | 943.4887 | +2 | 4254 |
| **LADTYTLYLKDTYTLYLK** | $y_{10}$iii + $y_8$iv | 1107.602 | +2 | 5416 |
| **ADTYTLYLKDTYTLYLK** | $y_9$iii + $y_8$iv | 1051.039 | +2 | 10267 |
| **ADTYTLYLKADTYTLYLK** | $y_9$iii + $y_9$iv | 1086.561 | +2 | 23404 |
| **LADTYTLYLKADTYTLYLK** | $y_{10}$iii + $y_9$iv | 1143.613 | +2 | 15266 |
| **LLADTYTLYLKDTYTLYLK** | iii +$y_8$iv | 1164.611 | +2 | 3689 |
| **LLADTYTLYLKADTYTLYLK** | iii +$y_9$iv | 1199.635 | +2 | 3074 |

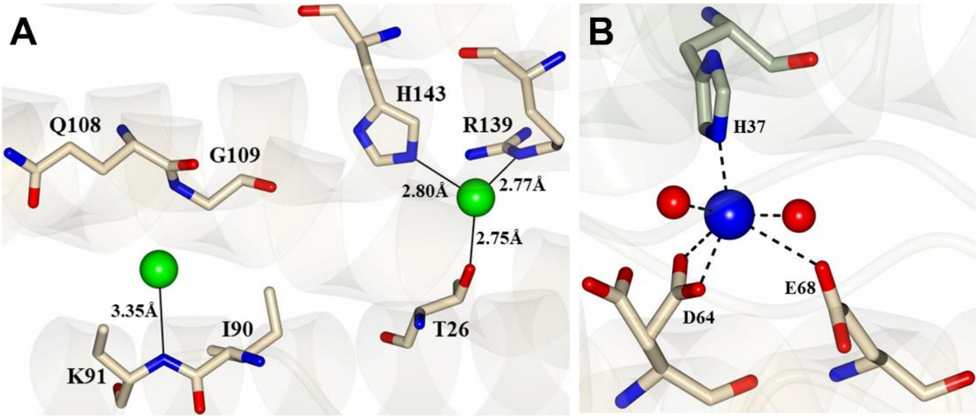


**Figure S1.** Chloride and sodium binding to Dps in the **Dps-o** structure. (**A**) Chloride (green spheres) and (**B**) sodium (blue sphere) bind to residues in **Dps-o** subunit dimers; water molecules (red spheres). D64 was modeled in alternate conformations in 5 of the 12 subunits.

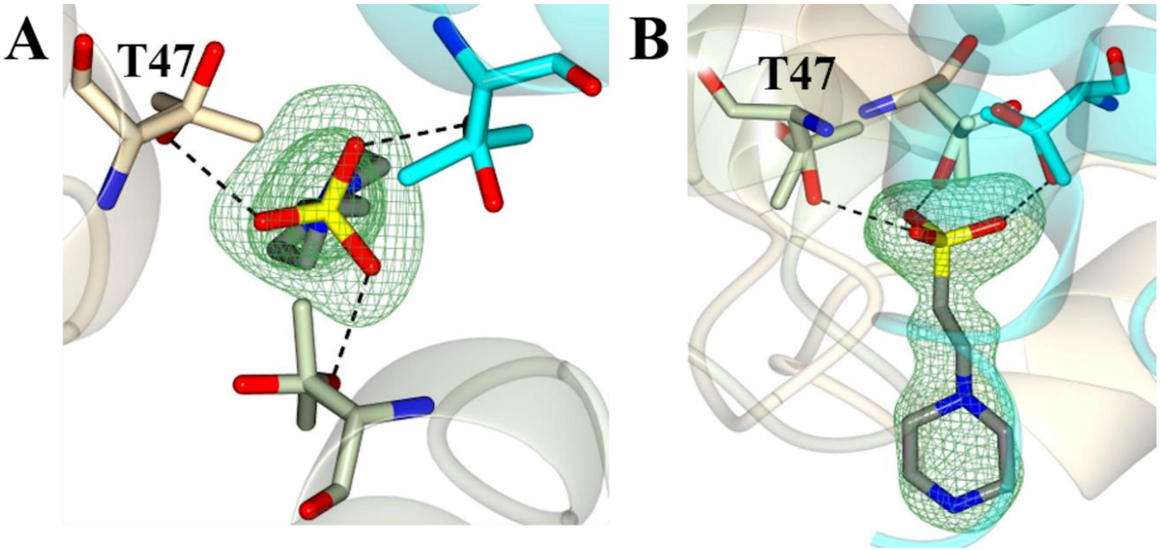


**Figure S2**. The type-A 3-fold pores are occupied by a HEPES buffer molecule. The Fo-Fc electron density (green mesh) is contoured at 3σ. The hydroxyethyl portion was disordered and could not be modeled. The views are (**A**) along and (**B**) perpendicular to the 3-fold axis.

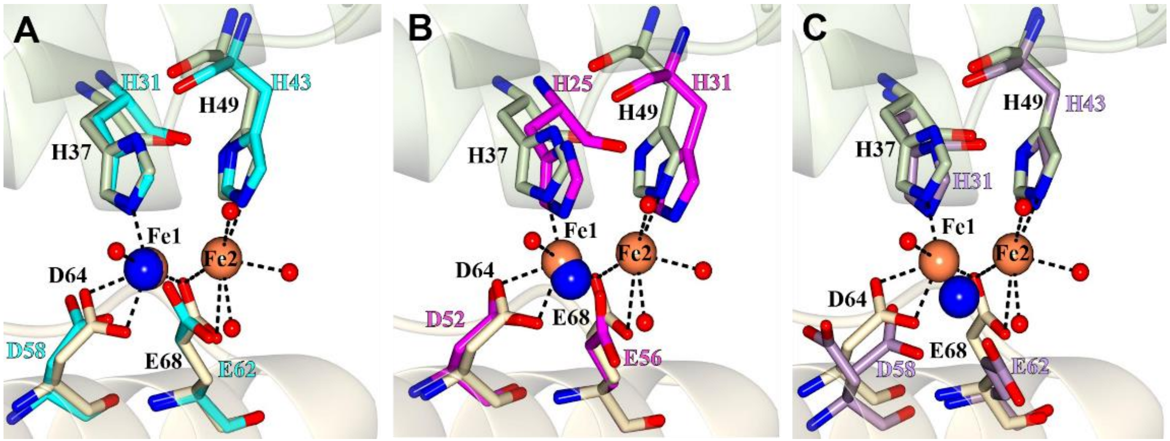


**Figure S3**. Superposition of Dps-c-Fe (wheat/green) with DPS homologs (A) *Listeria innocua* (cyan, 1QGH), (B) *Helicobacter pylori* (magenta, 1JI4) and (C) *Agrobacterium tumefaciens* (lilac, 1O9R) highlighting the ferroxidase center ligands. The iron ions in Dps-c-Fe and in the homologs are colored as orange and blue spheres, respectively. The coordinating residues for the homologs are indicated in their respective colors.

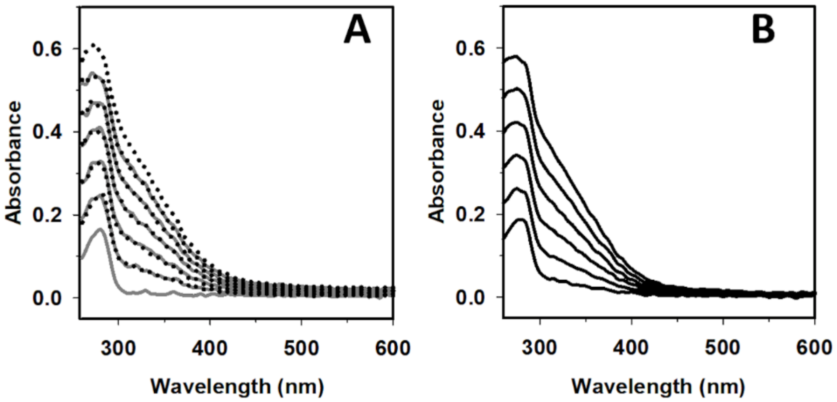


**Figure S4**. **(A) UV-vis spectra acquired during anaerobic mineralization of Pa Dps:** Pa Dps in 75 mM Bis-Tris (pH 6.5) containing 1 mM $Mg^{2+}$ was titrated in an anaerobic glove box, first with an aliquot of $Fe^{2+}$ delivering 50 $Fe^{2+}$/12-mer Pa Dps and then with an aliquot of delivering an equivalent of $H_2O_2$ relative to $Fe^{2+}$ until a total of 300 $Fe^{2+}$ ions/12-mer had been delivered. Note that the addition of an $Fe^{2+}$ aliquot does not bring changes to the spectra but the subsequent addition of an equivalent of $H_2O_2$ elicits an increase in the absorbance *ca*. 320 nm due to the formation of $Fe^{3+}$-O containing moieties. **(B) UV-vis spectra during mineralization of Pa Dps in air:** Note that the addition of each $Fe^{2+}$ aliquot brings an increase in the absorbance *ca*. 320 nm due to the formation of $Fe^{3+}$-O containing moieties.

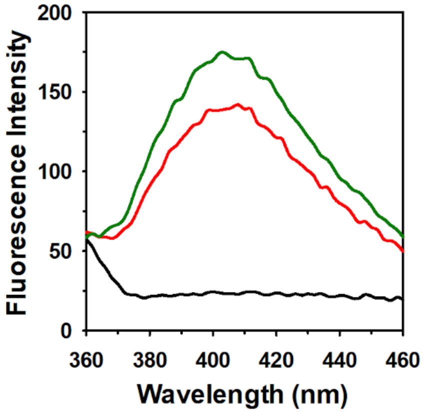


**Figure S5.** Fluorescence spectra (320 nm excitation) obtained during the titration of 12-mer Pa Dps (2.0 mL, 1.0 μM) in 75 mM Bis-Tris, pH 6.5 containing 1 mM $MgCl_2$ in an anaerobic glove box. The plots show the spectrum of Pa Dps before (black) and after the addition of two aliquots, each delivering 24 $Fe^{2+}$/12 mer and 1 equivalent of $H_2O_2$ (red and green, respectively).

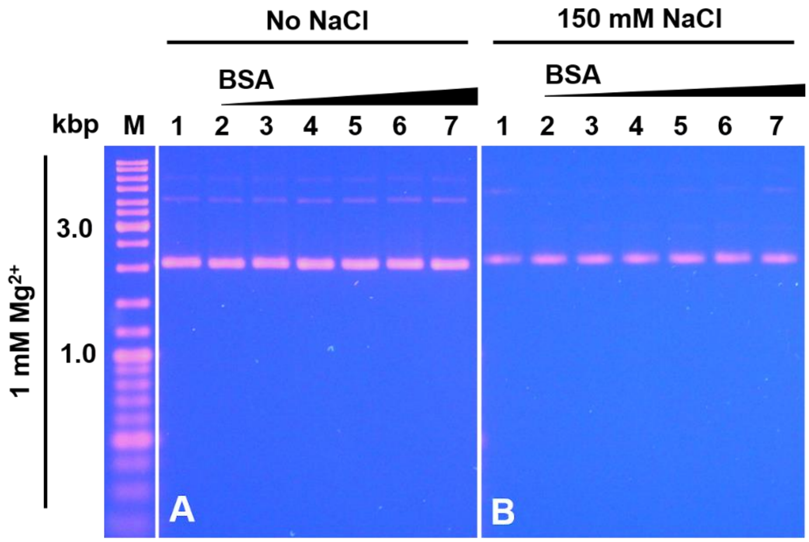


**Figure S6.** pUC18 plasmid DNA (8.6 nM) was incubated with distinct concentrations of bovine serum albumin (BSA) in (**A**) 75 mM Tris pH 7.5 containing 1 mM $MgCl_2$ or (B) 75 mM Tris pH 7.5 containing 150 mM NaCl and 1 mM $MgCl_2$. Lane 1 = DNA, lanes 2-7, respectively, BSA:DNA mole ratio 5, 10, 50, 100, 200 and 400. Lane M = DNA electrophoresis ladder.

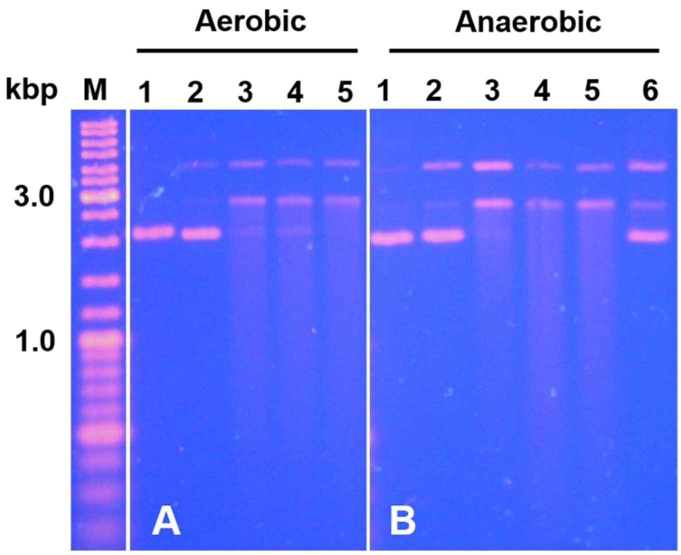


**Figure S7.** Pa Dps cleaves DNA under aerobic (**A**) or anaerobic (**B**) conditions. pUC18 plasmid DNA (8.6 nM) was incubated (1 h, 35 °C) with Pa Dps (Pa Dps:DNA mole ratio = 100) in 75 mM Tris pH 7.5, in the absence or presence of 1 mM divalent metal ions. Lane 1= DNA, lane 2-6, DNA and Pa DPS in the absence of divalent ion (2), and the presence of 1 mM $Mg^{2+}$ (3), $Mn^{2+}$ (4), $Ca^{2+}$ (5), or $Fe^{2+}$ (6).
